# Multidisciplinary administrative-professional-technical interventions to optimize antibiotic use and reduce resistance in a tertiary general hospital

**DOI:** 10.3389/fphar.2025.1592158

**Published:** 2025-09-19

**Authors:** Jingjing Han, Meiyu Shen, Yan Rao

**Affiliations:** ^1^ Department of Infection Control, Renmin Hospital of Wuhan University, Wuhan, Hubei, China; ^2^ Department of Psychiatry, Renmin Hospital of Wuhan University, Wuhan, Hubei, China; ^3^ Animal Biosafety Level III Laboratory at the Center for Animal Experiment, Wuhan University School of Medicine, Wuhan, Hubei, China

**Keywords:** antimicrobial stewardship, antimicrobial resistance, medical institution, multidisciplinary collaboration, antimicrobial management

## Abstract

**Introduction:**

Antimicrobial stewardship (AMS) is a cornerstone of global antimicrobial resistance (AMR) strategies. However, substantial differences exist in AMS implementation approaches and effectiveness among medical institutions across nations and regions. This study aimed to evaluate strategies focused on antimicrobial usage and AMR in a tertiary general hospital.

**Methods:**

This study was conducted in two phases: the baseline phase (January to June in 2023) and the intervention phase (July 2023 to June 2024). During the intervention phase, an innovative AMS strategy integrating multidisciplinary administrative, professional, and technical interventions was implemented to reduce antibiotic use and control antimicrobial resistance.

**Results:**

After intervention, the antimicrobial usage rate among inpatients decreased from 56.01% to 52.71% (*P* < 0.001). The antibiotic use density (AUD) decreased from 50.15 to 35.76 Defined Daily Doses (DDDs) per 100 patient-days (*P* < 0.001). The antimicrobial usage rates and AUD dropped significantly in medical, surgical, obstetrics and gynecology wards. Moreover, the intervention effect in pediatric wards displayed complex seasonal variation. The AUD for most antibiotic classes was significantly lower after the intervention. The detection rates of carbapenem-resistant *Klebsiella pneumoniae* and *Acinetobacter baumannii* decreased from 26.00% to 17.53% and 89.58% to 62.93%, respectively.

**Conclusion:**

The findings indicate that a multifaceted AMS strategy, integrating multidisciplinary administrative, professional, and technical interventions, offers a potentially effective strategy for reducing antimicrobial usage and combating AMR in the tertiary general hospital.

## Introduction

Antimicrobial resistance (AMR) is a global public health issue that affects human, animal, and environmental health (Velazquez-Meza et al., 2022). AMR limits the choice of treatment options, resulting in prolonged hospital stay and an increase in both mortality and medical costs (Pacios, 2022; Wozniak et al., 2022; Roberts et al., 2009). A study published by the Lancet on the global impact of AMR revealed a significant mortality burden, with more than 1.2 million people dying from drug-resistant infections in 2019, and indirectly causing an estimated 4.95 million deaths, exceeding fatalities from HIV/AIDS and malaria (Antimicrobial Resistance Collaborators, 2022). Projections suggest that if AMR is not controlled, 10 million people will die from drug-resistant infections annually by 2050, with a cumulative economic loss reaching $100 trillion (O'Neill, 2016). China is one of the largest consumers of antibiotics globally (Wagenlehner and Dittmar, 2022), and the prevalence of bacterial drug resistance is especially critical among *Enterobacteriaceae*, *Pseudomonas aeruginosa*, and *Acinetobacter* (Versporten et al., 2018). Urgent action, such as restricting antibiotic use, is needed to address AMR (Xiao, 2018).

Antimicrobial stewardship (AMS), introduced in 2007 by the Infectious Diseases Society of America (IDSA) and the Society for Healthcare Epidemiology of America (SHEA), is a cornerstone of global AMR strategies (World Health Organization, 2025). AMS improves the rational use of antibiotics, reduces AMR infections (Baur et al., 2017), and offers both medical and economic benefits (O'Donnell et al., 2022). Preventing the spread of resistant bacteria remains a global priority (Antimicrobial Resistance Collaborators, 2022; Nathwani et al., 2019). The World Health Organization (WHO), along with professional associations and governments, has promoted AMS programs to encourage the rational use of antimicrobials (Barlam et al., 2016). To support AMS implementation, WHO has provided guidance on the launch, structure, resources, strategy, and methodology for AMS (WHO, 2019). Despite WHO-led global efforts, challenges persist in healthcare settings, with limited evidence on the most effective and sustainable AMS interventions (Pulcini et al., 2019; Catho et al., 2022). Significant variations exist in AMS implementation models across different countries and regions. In developed countries, AMS typically adopts a technology-driven model centered around multidisciplinary team (MDT) comprising infectious disease specialists, clinical microbiologists, and specialized clinical pharmacists. While scientifically robust, this model heavily relies on both professional staffing and information system support, making it difficult to implement in regions with unevenly distributed medical resources. Medical institutions in developing countries like China face structural constraints including insufficient infectious disease specialists and underdeveloped teams of clinical pharmacists. Consequently, it primarily relies on administrative interventions such as hierarchical management of antimicrobial prescribing privileges and post-prescription review and feedback (Zhou and Ma, 2019). Although this approach yields rapid short-term results (National Health Commission of China, 2016-2018; Xiao et al., 2020), the lack of clinical technical support hinders the establishment of sustainable improvement mechanisms (Xiao, 2018; Zhou and Ma, 2019; Xiao et al., 2022). A comprehensive evaluation tool developed by the WHO revealed that while the national-level system and technical facilities for AMS in China are satisfactory, the system at the level of medical institutions needs to be strengthened (Xiao et al., 2022). Key areas that need to be improved include the construction of MDTs, implementation of professional intervention strategies, and enhanced professional education and outreach. The key challenge in current global AMS practice lies in developing scientific and feasible implementation models for resource-limited settings.

In this study, which was conducted at the Optics Valley branch of a tertiary teaching hospital in Wuhan, we demonstrate a pre-post evaluation of an innovative AMS intervention focused on antimicrobial usage and AMR. Through the collaborative leadership of the medical administration department and the infection control department, a novel AMS strategy integrating multidisciplinary administrative, professional, and technical interventions was developed.

## Methods

### Study location and subjects

This study was conducted at the Optics Valley branch of Renmin Hospital of Wuhan University from January 2023 to June 2024. The hospital is a large tertiary general hospital with three branches, including the Optics Valley branch, which has nearly 2,000 beds and covers medical wards, surgical wards, obstetrics and gynecology wards, pediatric wards, and the Intensive Care Unit (ICU). The study population consisted of patients hospitalized at the Optics Valley branch who were discharged between January 2023 and June 2024. The Clinical Research Ethics Committee of Renmin Hospital of Wuhan University approved this study (WDRY2024-K239) and waived the requirement for informed consent because of the retrospective nature of this study.

Inclusion criteria: 1) Inpatients discharged from the Optics Valley branch of Renmin Hospital of Wuhan University between January 2023 and June 2024. 2) Patients with complete inpatient records, including demographic information, hospitalization details, antimicrobial use, and microbial culture data.

Exclusion criteria: Patients with incomplete data necessary for the study.

### Study design and intervention

This study employed a retrospective quasi-experimental design. The 1.5-year study was divided into two phases: the baseline phase (from January to June in 2023) and the intervention phase (from July 2023 to June 2024). As the intervention was implemented hospital-wide, no parallel control group was established.

During the baseline phase, there were limited efforts to promote antimicrobial management through multidisciplinary collaboration at the Optics Valley branch of the hospital. It was characterized by insufficient data analysis and strategic guidance on antimicrobial use and resistant bacteria, inadequate training and education of medical staff on antimicrobial use, insufficient systems for data monitoring, prescription review, publicity, rewards, punishments, and feedback regarding antimicrobial management, insufficient computerized decision support, infrequent participation of clinical pharmacists in ward rounds, low pre-treatment microbiological specimen submission rate, and inadequate multidisciplinary collaboration in multidrug-resistant organism (MDRO) management.

In June 2023, with the support of hospital leadership, a comprehensive initiative to strengthen rational antimicrobial use was launched at the Optics Valley branch. From July 2023 to June 2024, an AMS strategy integrating multidisciplinary administrative, professional, and technical interventions, led by the infection control department and medical administration department, was developed.

1. Administrative management interventions:
(1) Multidisciplinary AMS team establishment: The infection management department and medical administration department set up a team to clarify the AMS goal with the support of clinical pharmacy, microbiology, infectious diseases, nursing and information center.
(2) Role allocation: The medical administration department conducted regular AMS meetings, monthly prescription reviews, target monitoring, and performance evaluations. The infection control department focused on improving pre-treatment microbiological specimen submission and reducing MDRO detection. Clinical pharmacists provided pharmaceutical support, while microbiologists analyzed resistance patterns. Nursing teams conducted health education and specimen collection, and infectious disease specialists participated in consultation process for complex infections and MDRO cases. The information center developed comprehensive monitoring, evaluation, and early warning systems.
(3) Policy and target setting: Antimicrobial-related policies were refined, and hospital- and ward-level antibiotic use density (AUD) targets were set, with rewards or penalties based on performance.
(4) Dynamic prescription management: A system for monitoring and penalizing inappropriate antimicrobial use was implemented monthly, including retraining and point deductions for prescription rights.
(5) Education and training: The AMS team conducted quarterly hospital-wide training (online and in-person) on rational antimicrobial use, antimicrobial resistance patterns, and MDRO management.
(6) MDRO control: A multidisciplinary MDRO team led by the infection control department strengthened MDRO monitoring, management, and training, through quarterly reports and collaborative interventions.
2. Technical interventions:
(1) Electronic prescription governance: a)Strengthen the automated authorization system requiring senior physician approval and pharmacist review for special-class agents. b)Computerized decision support was implemented, featuring real-time alerts for surgical prophylaxis duration (24 h for Class 0/I, 48 h for Class II, and 7days for Class III procedures). The system automatically flags therapeutic antimicrobial courses exceeding 7 days, requiring physician evaluation for subsequent 7-day extensions, and mandatory infectious disease consultation for total durations >14 days.
(2) Diagnostic stewardship protocols: a)Mandatory pre-treatment microbiological specimen submission, including ≥50% for standard antimicrobial therapy, and 100% for high-priority combinations (carbapenems, glycopeptides, β-lactam/β-lactamase inhibitors, etc.). b)Enhanced critical value notification for positive blood cultures.
(3) Antimicrobial selection software: a)Therapeutic drug monitoring program. b)Mobile decision support application.
3. Professional interventions:
(1) Clinical pharmacist integration: Infectious disease pharmacists conducted daily ICU rounds and participated in complex case discussions.
(2) Multidisciplinary management: We strengthened structured consultations for complex or MDRO infections.
(3) Data-driven stewardship: a)Monthly antimicrobial prescription reviews by infectious disease specialists and clinical pharmacists. b)Quarterly reports analyzing hospital-acquired infection rates, and MDRO trends. c)Regular multidisciplinary meetings to analyze prescribing trends, evaluate intervention effectiveness, and refine stewardship strategies.

### Definition

The value of the Defined Daily Dose (DDD) for antibacterial drugs was determined according to WHO guidelines and the daily dose recommended in drug instructions (Who Collaborating Centre For Drug Statistics Methodology, 2023). It is calculated as: DDD = total dosage of a drug (g)/DDD value of the drug. The cumulative DDDs of antibiotics for inpatients represents the sum of all antibiotic DDDs. AUD is defined as the DDD of antibiotics consumed per 100 patient-days. It is calculated as: AUD = cumulative DDDs of antibiotics*100/(The number of patients who were treated during the same period*Average days in hospital). The antimicrobial usage rate refers to the proportion of patients who received systemic antibiotics relative to the total number of patients in the same period. Only the antibiotic prescriptions for systemic administration were included in the analysis.

The MDRO detection rate is calculated as: number of strains identified as MDRO/total number of strains detected from the same pathogens during the same period×100%. MDRO include Methicillin-resistant *Staphylococcus aureus* (MRSA), Vancomycin-resistant *Enterococcus* (VRE), and carbapenem-resistant *Enterobacteriaceae* (CRE), such as carbapenem-resistant *Escherichia coli* (CREC), carbapenem-resistant *Klebsiella pneumoniae* (CRKP), carbapenem-resistant *Acinetobacter baumannii* (CRAB), and carbapenem-resistant *Pseudomonas aeruginosa* (CRPA).

### Bacterial identification and susceptibility testing

Quality control strains used included *Escherichia coli* ATCC25922, *Pseudomonas aeruginosa* ATCC27853, *Staphylococcus aureus* ATCC25923, *Staphylococcus aureus* ATCC29213, *Enterococcus faecalis* ATCC29212, and *Streptococcus pneumoniae* ATCC49619. Strain identification was conducted using the Merieux VITEK MS identification mass spectrometer, while drug sensitivity testing was performed using the Merieux VITEK 2 COMPACT automated microbial analysis system. Repeated strains isolated from the same site in the same patient were excluded from the statistical analysis of clinically isolated pathogens.

### Data collection

Patient data, including age, gender, bed number, hospitalization number, admission/discharge date, ward, diagnosis, microbial culture results (pathogen name, and multidrug-resistant status), and antibiotic usage, were collected for hospitalized patients discharged between January 2023 and June 2024. Data were extracted from the Xinglin Hospital Infection Information System, integrating hospital information system and laboratory information system. Antibiotic usage data, Including information such as drug name, dosage form, antimicrobial category, number of patients treated, and DDDs, were obtained from the Yiyao Rational Drug Use System, covering hospital-wide and ward-specific usage.

### Classification definition

1. Ward classification: The surgical wards include all wards primarily focused on surgical procedures, except for obstetrics and gynecology ward, encompassing thoracic surgery, orthopedics, ophthalmology, gastrointestinal surgery, hepatobiliary surgery, neurosurgery, urology, breast and thyroid surgery, otolaryngology, and vascular surgery. The obstetrics and gynecology wards comprise both obstetrics wards and gynecology wards, while the pediatric wards include the pediatric ward and neonatal ward. For simplicity, in addition to traditional medical wards like respiratory, gastroenterology, cardiology, endocrinology, neurology, nephrology, and oncology, non-surgical wards such as rehabilitation, traditional chinese medicine, dermatology, and psychiatry were also included for antibiotic statistics.
2. Antibiotic classification: Antibiotics were classified based on the WHO ATC/DDD system (ATC/DDD Index, 2024): penicillins (J01C), first-generation cephalosporins (J01DB), second-generation cephalosporins (J01DC), third-generation cephalosporins (J01DD), carbapenems (J01DH), quinolones (J01M), macrolides (J01F), aminoglycosides (J01G), glycopeptide antibiotics and linezolid (J01XA + J01XX08), imidazole derivatives (J01XD), antifungal drugs (J02AC), and others including tetracyclines (J01A), polymyxins (J01XB), fosfomycin (J01XX01), and nitrofuran derivatives (J01XE).

### Bias control measures

1. Selection bias control:
- Inclusion criteria validation: Random sample verification confirmed no critical data omissions in included cases.
2. Information bias prevention:
- Data sources: Implemented linkage between the hospital electronic medical record system and pharmacy databases to ensure data completeness.
- Data extraction quality control: Conducted via a four-phase process: training, trial extraction, formal extraction, and verification.
- Quarterly data specification: Quarterly antimicrobial usage data were automatically aggregated by the hospital pharmacy system and validated through monthly audits by the pharmacy department.
3. Key variable definitions:
- Resistant pathogen determination: Strictly adhered to the guidelines of the Clinical & Laboratory Standards Institute (CLSI).

### Statistical analysis

SPSS 22.0 (IBM Corp., Armonk, NY, United States) was used for statistical analysis. Measurement data were tested for normality using the Kolmogorov-Smirnov method (sample size ≥ 50) or the Shapiro-Wilk method (sample size < 50). Normally-distributed data are described as the mean ± standard deviation (SD) and were compared between groups using the t-test for independent samples. Non-normally-distributed data are presented as median (interquartile range [IQR]) and were analyzed using the Mann–Whitney U test. Count data are shown as frequency (percentage) and were compared between groups using the chi-squared test or Fisher’s exact test. Incidence rates before and after intervention were compared using unadjusted relative risk (RR), calculated as the rate of events within each time period. To account for potential confounding by seasonal variations, we conducted additional analyses comparing data from corresponding quarters pre- and post-intervention. Stratified analyses were performed by ward level and different types of antibiotics. GraphPad Prism 9 (GraphPad Software, San Diego, CA, United States)was used for graphical representations. *P* < 0.05 was considered statistically significant.

## Results

### Study population and baseline characteristics

Inpatients discharged before and after the intervention were included as study subjects. No significant differences in gender or age were observed between the pre- and post-intervention groups (*P* > 0.05). See Table 1 for details.

**TABLE 1:** Basic information of the subjects before and after intervention.

Characteristic	Baseline (n = 31,342)	Intervention (n = 71,832)	P Value
Gender, n (%)			0.564
Male	15,847 (50.56%)	36,179 (50.37%)	
Female	15,495 (49.44%)	35,653 (49.63%)	
Age (year), n (%)			0.090
≥65	8311 (26.52%)	18,685 (26.01%)	
<65	23,031 (73.48%)	53,147 (73.99%)	
Number of patients per month, median (IQR)	5637 (4594,5808)	6192 (5863,6385)	0.013
Patients in different wards, n (%)			<0.001
Medical Ward	12,940 (41.29%)	29,452 (41.00%)	
Surgery Ward	13,000 (41.48%)	28,597 (39.81%)	
Gynecology and Obstetric Ward	2319 (7.40%)	4,832 (6.73%)	
Pediatric (including neonatology) Ward	2977 (9.50%)	8768 (12.21%)	
ICU	106 (0.34%)	183 (0.25%)	
Patient-days per month	49,457.50 ± 6379.42	58691.50 (56041.50,62148.50)	0.010

ICU, Intensive Care Unit; IQR, interquartile range.

### Overall antimicrobial usage

**FIGURE 1:**
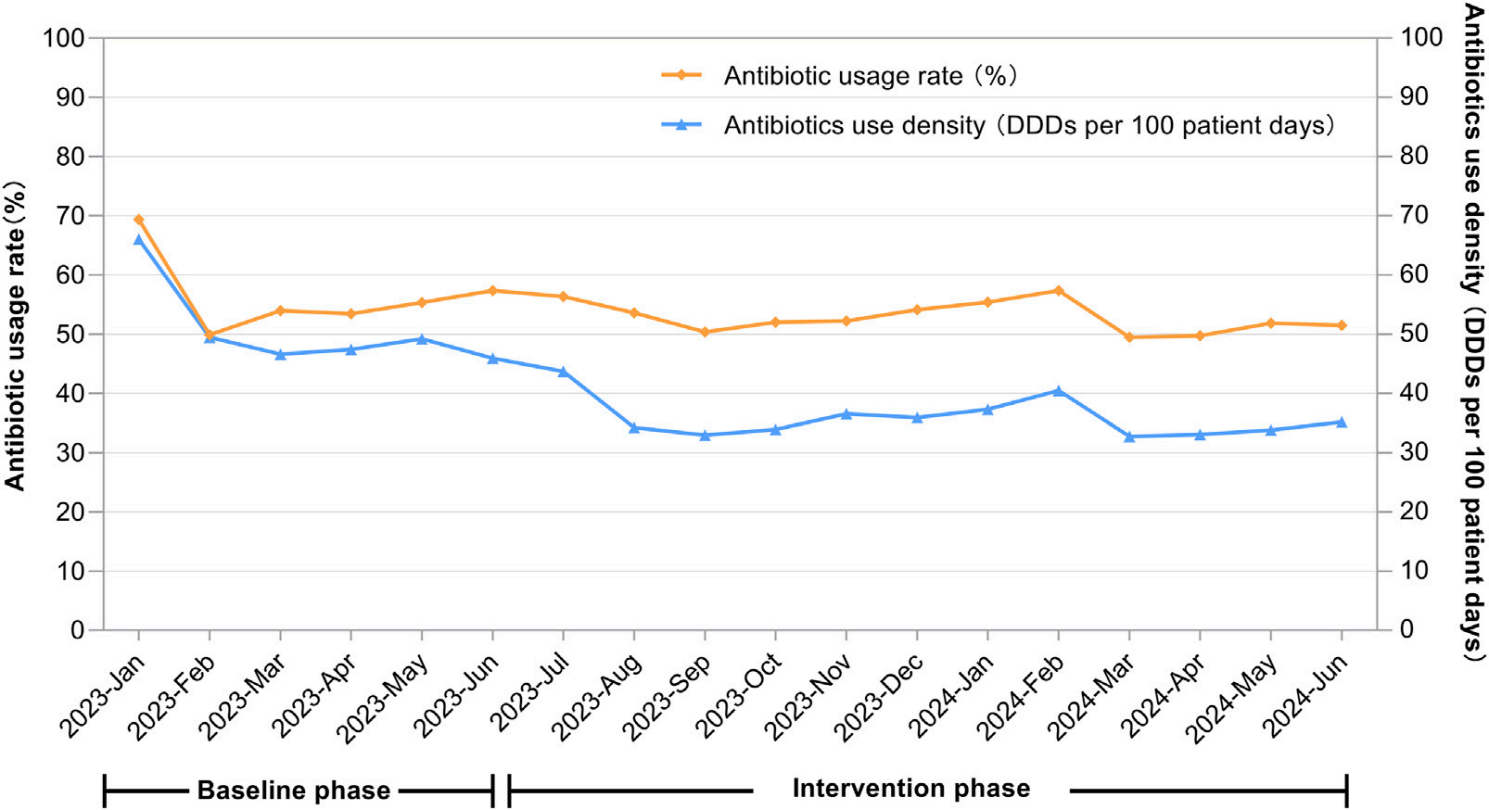
Temporal changes in the antibiotic usage rate (%) and antibiotics use density (DDDs per 100 patient-days) across the entire hospital.

After the intervention, the AUD decreased from 50.15 to 35.76 DDDs/100 patient-days (*P* < 0.001), and the antimicrobial usage rate dropped from 56.01% to 52.71% (*P* < 0.001). After controlling for seasonal variations through quarter-to-quarter comparisons, both the antimicrobial usage rate and AUD remained significantly lower during the intervention period compared to baseline. Specific data are provided in Table 2 and Figure 1.

**TABLE 2:** Comparison of indicators related to antimicrobial use in the whole hospital before and after intervention.

Characteristic	Primary analysis				Quarterly matched comparison							
	Baseline (2023.1–6)	Intervention (2023.7–2024.6)	RR (95% CI)	P Value	Baseline (2023-Q1)	Intervention (2024-Q1)	RR (95% CI)	P Value	Baseline (2023-Q2)	Intervention (2024-Q2)	RR (95% CI)	P Value
Patients using antibiotics	17,556	37,862			8099	8871			9,457	9,499		
Number of patients	31,342	71,832			14,261	16,550			17,081	18,628		
Number of patient-days (patient-days)	296,745	692,784			135,975	155,609			160,770	170,020		
Cumulative DDDs	148,815.85	247,746.64			72,508.65	56,631.79			76307.20	57750.80		
Antimicrobial usage rate (%)	56.01	52.71	0.941 (0.930–0.952)	<0.001	56.79	53.60	0.944 (0.925–0.963)	<0.001	55.36	50.99	0.921 (0.903–0.939)	<0.001
Antibiotics use density (DDDs/100 patient-days)	50.15	35.76	0.713 (0.710–0.717)	<0.001	53.32	36.39	0.682 (0.677–0.688)	<0.001	47.46	33.97	0.716 (0.710–0.722)	<0.001

DDD, Defined Daily Dose; RR, relative risk.

### Ward-level usage

**FIGURE 2:**
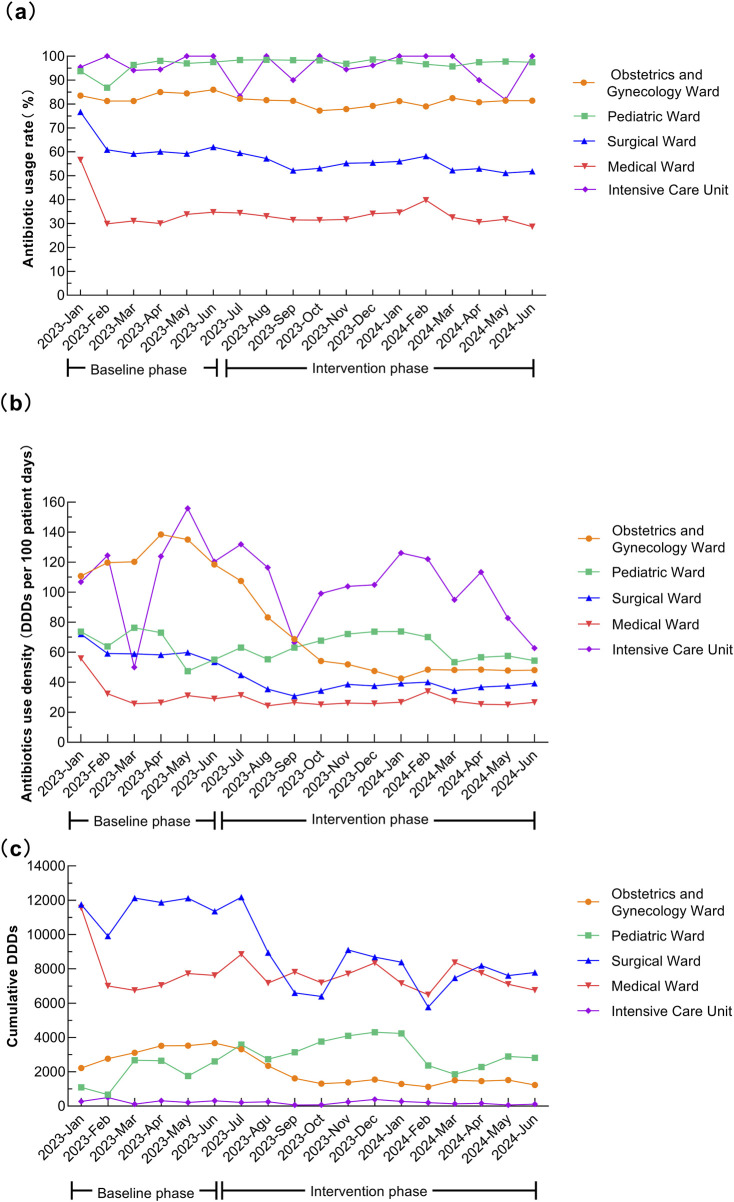
Temporal trends in antibiotic usage across different hospital wards. **(a)** Antibiotic usage rate (%). **(b)** Antibiotics use density (DDDs per 100 patient-days). **(c)** Cumulative DDDs.

After the intervention, the antimicrobial usage rate in medical wards decreased from 35.15% to 32.67% (*P* < 0.001), in surgical wards from 62.28% to 54.57% (*P* < 0.001), and in obstetrics and gynecology wards from 83.66% to 80.52% (*P* = 0.001). The AUD for medical wards decreased from 32.57 to 26.80 DDDs/100 patient-days (*P* < 0.001), for surgical wards from 59.86 to 37.51 DDDs/100 patient-days (*P* < 0.001), and for obstetrics and gynecology ward from 124.15 to 58.12 DDDs/100 patient-days (*P* = 0.001). In the quarter-matched comparison, the medical and surgical wards maintained significantly lower antimicrobial usage rates and AUD during the intervention period. Q2 of 2024 exhibited significant reductions in both antimicrobial usage rate and AUD in obstetrics and gynecology ward. Pediatric ward displayed complex seasonal variation. Q2 data of ICU revealed selective AUD improvement without corresponding rate change after intervention. Specific data are provided in Table 3 and Figure 2.

**TABLE 3:** Comparison of indicators related to antimicrobial use in different wards before and after intervention.

Subgroup/Period	Characteristic	Medical ward				Surgery ward			
		Baseline	Intervention	RR(95%CI)/t/Z	*P* Value	Baseline	Intervention	RR(95%CI)/t/Z	*P* Value
Primary Analysis	Antimicrobial usage rate (%)	35.15 (4,548/12,940)	32.67 (9,622/29,452)	0.930 (0.903–0.956)	<0.001	62.28 (8097/13,000)	54.57 (15,605/28,597)	0.876 (0.861–0.891)	<0.001
	Antibiotics use density (DDDs/100 patient-days)	32.57	26.80	0.823 (0.815–0.831)	<0.001	59.86	37.51	0.627 (0.622–0.631)	<0.001
	Antibiotics use density of every month (DDDs/100 patient-days)	30.03 (26.33, 32.36)	26.32 (25.23, 27.06)	-1.592	0.125	60.30 ± 6.24	37.42 ± 3.51	10.065	<0.001
Quarterly Matched Comparison(2023-Q1 VS 2024-Q1)	Antimicrobial usage rate (%)	37.80 (2294/6,069)	35.00 (2427/6934)	0.926 (0.885–0.969)	0.001	64.47 (3,868/6,000)	55.10 (3,538/6,421)	0.855 (0.830–0.880)	<0.001
	Antibiotics use density (DDDs/100 patient-days)	36.92	28.80	0.780 (0.768–0.792)	<0.001	63.02	37.58	0.596 (0.589–0.604)	<0.001
Quarterly Matched Comparison(2023-Q2 VS 2024-Q2)	Antimicrobial usage rate (%)	32.80 (2254/6,871)	30.42 (2325/7,642)	0.927 (0.884–0.973)	0.002	60.41 (4,229/7,000)	51.98 (3,800/7,310)	0.860 (0.836–0.886)	<0.001
	Antibiotics use density (DDDs/100 patient-days)	28.74	25.61	0.891 (0.877–0.906)	<0.001	57.12	37.90	0.663 (0.655–0.672)	<0.001

Gynecology and obstetrics ward				Pediatric (including neonatology) ward				ICU			
Baseline	Intervention	RR(95%CI)/t/Z	*P* Value	Baseline	Intervention	RR(95%CI)/t/Z	*P* Value	Baseline	Intervention	RR(95%CI)/t/Z	*P* Value
83.66 (1940/2,319)	80.52 (3,891/4,832)	0.963 (0.941–0.985)	0.001	96.34 (2868/2977)	97.74 (8570/8768)	1.015 (1.007–1.022)	<0.001	97.17 (103/106)	95.08 (174/183)	0.979 (0.934–1.025)	0.391
124.15	58.12	0.581 (0.576–0.586)	0.001	63.24	63.99	1.012 (0.999–1.025)	0.067	112.54	104.87	-	-
123.79 ± 10.64	48.42(47.98, 61.52)	-3.372	<0.001	64.90 ± 11.63	63.42 ± 7.89	0.322	0.751	113.59 ± 35.05	102.07 ± 22.28	0.855	0.405
81.86 (862/1,053)	81.07 (985/1,215)	0.990 (0.952–1.030)	0.629	94.27 (1,020/1,082)	96.94 (1868/1927)	1.028 (1.011–1.046)	<0.001	96.49 (55/57)	100.00 (53/53)	1.036 (0.986–1.089)	0.496
117.34	46.23	0.462 (0.452–0.473)	0.001	73.52	67.20	0.914 (0.896–0.932)	<0.001	100.20	116.48	-	-
85.15 (1,078/1,266)	81.06 (1,036/1,278)	0.952 (0.919–0.986)	0.006	97.52 (1848/1895)	97.59 (2307/2364)	1.001 (0.991–1.010)	0.885	97.96 (48/49)	91.18 (31/34)	0.931 (0.832–1.041)	0.300
129.85	48.14	0.481 (0.471–0.492)	<0.001	58.11	56.15	0.966 (0.946–0.987)	0.001	129.17	84.78	0.847 (0.813–0.882)	<0.001

ICU, Intensive Care Unit; DDD, Defined Daily Dose; RR, relative risk.

### Use of different antibiotic classes

**FIGURE 3:**
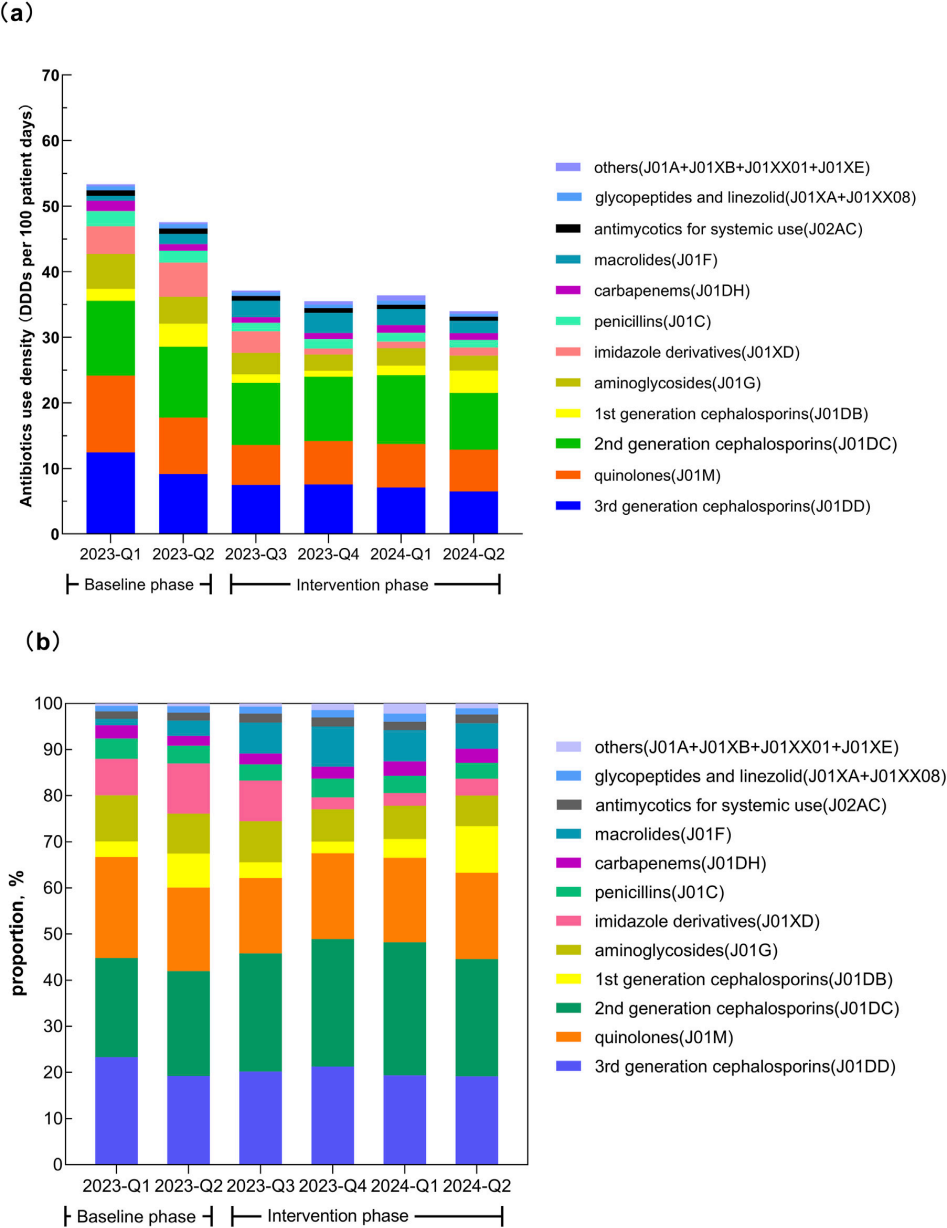
Temporal changes in the consumption of different classes of antibiotics. **(a)** Antibiotics use density (DDDs per 100 patient-days). **(b)** Proportion of total consumption.

Except for macrolides, the AUD for all other antibiotic classes was significantly lower after the intervention (all *P* < 0.001). After the intervention, the top five antibiotic classes ranked by AUD were second-generation cephalosporins, third-generation cephalosporins (including enzyme inhibitor compound preparations), quinolones, aminoglycosides, and macrolides, accounting for 79.31% of total usage. In the quarter-matched comparison, significantly lower AUD values were maintained for most antimicrobial classes including third-generation cephalosporins, quinolones, second-generation cephalosporins, aminoglycosides, nitroimidazoles, penicillins, and antifungals during the intervention period compared to baseline. The exception was macrolides, which showed persistently higher AUD. Notably, three classes exhibited seasonal variation: glycopeptides and linezolid, first-generation cephalosporins and carbapenems. See Table 4 and Figure 3 for details.

**TABLE 4:** Comparison of the antibiotics use density of different antibacterial drugs before and after intervention.

Antibiotic class	Antibiotics use density (DDDs/100 patient-days)				Quarterly matched comparison-antibiotics use density (DDDs/100 patient-days)							
	Baseline	Intervention	RR (95% CI)	P Value	Baseline (2023-Q1)	Intervention (2024-Q1)	RR (95% CI)	P Value	Baseline (2023-Q2)	Intervention (2024-Q2)	RR (95% CI)	P Value
3rd cephalosporins	10.66	7.16	0.672 (0.663–0.681)	<0.001	12.44	7.06	0.567 (0.554–0.580)	<0.001	9.14	6.50	0.710 (0.694–0.728)	<0.001
quinolones	10.02	6.41	0.640 (0.631–0.649)	<0.001	11.69	6.67	0.570 (0.557–0.584)	<0.001	8.60	6.36	0.739 (0.721–0.757)	<0.001
2nd cephalosporins	11.11	9.60	0.865 (0.854–0.875)	<0.001	11.45	10.49	0.916 (0.898–0.936)	<0.001	10.82	8.64	0.799 (0.782–0.816)	<0.001
1st cephalosporins	2.73	1.75	0.641 (0.623–0.659)	<0.001	1.79	1.47	0.824 (0.779–0.872)	<0.001	3.52	3.43	0.973 (0.938–1.008)	0.130
aminoglycosides	4.67	2.69	0.575 (0.563–0.588)	<0.001	5.34	2.64	0.494 (0.475–0.512)	<0.001	4.10	2.26	0.552 (0.531–0.574)	<0.001
nitroimidazoles	4.73	1.64	0.346 (0.338–0.354)	<0.001	4.20	0.99	0.236 (0.223–0.249)	<0.001	5.18	1.23	0.237 (0.226–0.248)	<0.001
penicillins	2.08	1.33	0.641 (0.621–0.662)	<0.001	2.35	1.38	0.585 (0.554–0.618)	<0.001	1.84	1.17	0.636 (0.602–0.673)	<0.001
carbapenems	1.26	0.98	0.778 (0.747–0.809)	<0.001	1.55	1.14	0.733 (0.689–0.781)	<0.001	1.01	1.03	1.023 (0.956–1.094)	0.514
macrolides	1.20	2.50	2.075 (2.002–2.150)	<0.001	0.76	2.47	3.269 (3.053–3.501)	<0.001	1.58	1.88	1.190 (1.130–1.253)	<0.001
antifungals	0.82	0.68	0.836 (0.797–0.878)	<0.001	0.82	0.66	0.804 (0.739–0.874)	<0.001	0.81	0.64	0.787 (0.726–0.852)	<0.001
glyeptides and linezolid	0.65	0.56	0.851 (0.806–0.898)	<0.001	0.66	0.64	0.979 (0.894–1.071)	0.638	0.65	0.46	0.713 (0.650–0.782)	<0.001

DDD, Defined Daily Dose; RR, relative risk.

### MDRO trends

After the intervention, the detection rates of CRKP and CRAB decreased from 26.00% to 17.53% and 89.58% to 62.93%, respectively (both *P* < 0.05). The detection rate of CRPA was 30.43% before the intervention and decreased afterward, though it was not statistically significant (*P* = 0.734). After controlling for seasonal variations through quarter-to-quarter comparisons, the first intervention quarter showed the detection rates of CRKP and CRAB remained significantly lower than those in the baseline period. Although a decreasing trend was observed in the second quarter, the difference did not reach statistical significance. See Table 5 for details.

**TABLE 5:** Comparison of detection rates of multidrug-resistant organisms before and after the intervention.

Item	Primary analysis				Quarterly matched comparison							
	Baseline	Intervention	χ2	P Value	Baseline (2023-Q1)	Intervention (2024-Q1)	χ2	P Value	Baseline (2023-Q2)	Intervention (2024-Q2)	χ2	P Value
Detection of MRSA (%)	27.66 (26/94)	21.97 (67/305)	1.303	0.254	38.00 (19/50)	27.27 (24/88)	1.710	0.191	15.91 (7/44)	15.85 (13/82)	0.000	0.994
Detection of VRE(%)	0 (0/211)	0 (0/418)	-	-	0 (0/99)	0 (0/72)	-	-	0 (0/112)	0 (0/120)	-	-
Detection of CREC (%)	1.98 (5/253)	1.68 (11/656)	0.095	0.758	0 (0/121)	2.16 (3/139)	–	0.251^a^	3.79 (5/132)	1.33 (2/150)	-	0.258^a^
Detection of CRKP(%)	26.00 (39/150)	17.53 (64/365)	4.762	0.029	28.33 (17/60)	14.63 (12/82)	4.001	0.045	24.44 (22/90)	16.87 (14/83)	1.504	0.220
Detection of CRAB (%)	89.58 (43/48)	62.93 (73/116)	11.650	0.001	100.00 (25/25)	70.00 (21/30)	8.967	0.003	78.26 (18/23)	66.67 (18/27)	0.828	0.363
Detection of CRPA (%)	30.43 (21/69)	28.36 (76/268)	0.115	0.734	30.77 (8/26)	35.00 (21/60)	0.145	0.703	30.23 (13/43)	22.22 (12/54)	0.803	0.370

^a^ Fisher’s exact probability method. MRSA, Methicillin-resistant Staphylococcus aureus; VRE, Vancomycin-resistant Enterococcus; CREC, carbapenem-resistant Escherichia coli; CRKP, carbapenem-resistant Klebsiella pneumoniae; CRAB, carbapenem-resistant Acinetobacter baumannii; CRPA, carbapenem-resistant Pseudomonas aeruginosa.

### Training and auditing of medical staff

The Optics Valley branch, with approximately 1,300 medical staff, conducted six hospital-wide training sessions during the intervention period, covering antimicrobial use, antimicrobial resistance patterns, and MDRO. These sessions reached 5,056 participants, including 1,504 doctors, 3,258 nurses, and 294 pharmacists and administrative staff.

The medical administration department organized monthly prescription reviews of inpatient antimicrobial use, reviewing approximately 100 records each month. Cases of inappropriate use were reported hospital-wide, with retraining for relevant staff. In July 2023, 10 cases of inappropriate use were identified, primarily involving unjustified use, prolonged treatment, excessive dosage, and non-standardized combinations. By July 2024, this number decreased to two cases, mainly characterized by prolonged treatment and excessive dosage.

## Discussion

In this study, we employed a retrospective quasi-experimental design to evaluate the effectiveness of an innovative AMS strategy integrating multidisciplinary administrative, professional, and technical interventions in reducing antimicrobial use and resistant pathogen detection. This intervention reduced antimicrobial usage rates and AUD, as well as detection rates of carbapenem-resistant Gram-negative bacteria, such as CRKP and CRAB.

Before the intervention, the antimicrobial usage rates in our hospital, particularly in surgical wards and ICU, were higher than those reported in previous study (Xiao et al., 2023a). The overall usage rate decreased to 52.71%, and AUD dropped to 35.76 DDDs/100 patient-days after intervention. After controlling for seasonal variations through quarter-to-quarter comparisons, both the antimicrobial usage rate and AUD remained significantly lower during the intervention period compared to baseline, suggesting robust intervention effects independent of seasonal factors. Both the antimicrobial usage rate and AUD in medical and surgical wards significantly decreased after intervention, falling below levels reported in previous studies (Xiao et al., 2023a; Xiao et al., 2023b). However, the ICU and obstetrics and gynecology ward still showed higher usage rates, indicating a need for further action. In pediatric ward (including neonates), the antimicrobial usage rate before and after intervention remained higher than in previous studies (Wang et al., 2021). Comparative analysis of AUD by wards in seasonally matched periods revealed distinct patterns. The medical and surgical wards maintained significantly lower antimicrobial usage rates and AUD during the intervention period, suggesting limited seasonal confounding effects. The obstetrics and gynecology ward demonstrated differential seasonal patterns: while Q1 showed non-significant rate reduction but significant AUD decrease, Q2 exhibited significant reductions in both usage rate and AUD. Pediatric ward displayed complex seasonal variation, indicating substantial seasonal modulation. The ICU cohort showed no significant overall reductions. However, Q2 data revealed selective AUD improvement without corresponding rate change, suggesting initial positive intervention effects. The data revealed significant ward-specific patterns, underscoring the need for targeted interventions. Notably, pediatric ward demonstrated strong seasonal fluctuations in antimicrobial use, likely driven by epidemic-prone infections (e.g., respiratory viruses and *mycoplasma* pneumonia). It suggests that a dynamic and seasonally adapted AMS strategies such as intensified stewardship during high-incidence periods could optimize management of antimicrobial use.

In this study, the use of most other classes of antibiotics except for macrolides decreased after the intervention. The AUD of third-generation cephalosporins (including enzyme inhibitor combinations), quinolones, carbapenems, and other antibiotics were lower than the national average (National Health Commission of China, 2016-2018). Comparative analysis of AUD by classes of antibiotics in seasonally matched periods revealed distinct patterns. During the intervention period, significantly lower AUD values were maintained for most antimicrobial classes, suggesting minimal seasonal confounding. The exception was macrolides, which showed persistently higher AUD, mainly azithromycin. Peak usage of macrolides (azithromycin) occurred in Q3-Q4 2023, with sustained elevation in Q1-Q2 2024 (though slightly moderated) compared to pre-intervention levels. The increase directly correlated with the Mycoplasma pneumoniae pneumonia (MPP) outbreak in Wuhan in Q3-Q4 2023. Although no outbreak occurred in 2024, MPP incidence remained elevated in Q1-Q2 2024 compared to 2023 baseline. There was a significant rise in pediatric hospitalizations. Stable azithromycin use in obstetrics and general surgery confirms the respiratory infection-driven pattern. Notably, three classes exhibited seasonal variation. Glycopeptides and linezolid demonstrated non-significant reduction in Q1 but significant decrease in Q2 (consistent with overall trend), while both first-generation cephalosporins and carbapenems showed significant Q1 reductions but non-significant Q2 changes. These differential quarterly patterns suggest potential seasonal influences on these three antimicrobial classes. Before the intervention, the detection rate of CRKP was 26.00%, and CRAB was 89.58%, both higher than the national average (National Health Commission of China, 2016-2018). After the intervention, the detection rates dropped to 17.53% for CRKP and 62.93% for CRAB. When comparing identical quarters between intervention and baseline periods, the first intervention quarter showed the detection rates of CRKP and CRAB remained significantly lower than those in the baseline period. Although a decreasing trend was observed in the second quarter, the difference did not reach statistical significance, suggesting that seasonal factors might influence MDRO detection rates, possibly due to insufficient sample size. Additionally, the lack of a significant reduction in carbapenem consumption during the second quarter of 2024 may be associated with the persistent detection of CRKP and CRAB, indicating a potential correlation between antimicrobial use and resistance patterns. Considering that the third and fourth quarters of 2023 represented the early or transitional phase of intervention implementation, and these two-quarters coincided with the peak season for respiratory infectious diseases in Wuhan (with a high incidence of *mycoplasma* pneumonia in Q3 and influenza in Q4 2023), the results remained robust after excluding the impact of these two-quarters.

This study innovatively develops an AMS strategy integrating multidisciplinary administrative, professional, and technical interventions. The AMS intervention features three innovations:1) Management innovation: Contrasting with western reliance on MDTs with weak administrative constraints and China’s traditional administrative-command approach lacking technical support, we established an AMS framework equally emphasizing administrative authority and technical and professional expertise. 2) Professional and technical interventions in our study exhibit both convergence with and divergence from established AMS programs in Europe and North America. Notable alignments include electronic prescription governance systems, diagnostic stewardship protocols, and multidisciplinary collaboration, while key distinctions are a stratified pre-treatment microbiological specimen submission protocol (50%/100%) tailored to resource-constrained settings, and mobile decision support application addressing infrastructure gaps. This framework uniquely integrates administrative, professional, and technical interventions with institutional hierarchies. 3) Implementation pathway innovation: We constructed a matrix AMS organizational structure combining vertical management with horizontal collaboration. Vertically, a leadership team (medical administration department and infection control department) sets policies, allocates resources, and supervises implementation; horizontally, an MDT team provides technical support. Notably, we elevated infection control to equal decision-making status with medical administration, breaking departmental barriers through joint directives and cross-evaluation. This “administration-clinical-infection control” collaborative model embodies the systemic nature of AMS. Multiple lines of evidence suggest the observed improvements may be attributed to the intervention: trend analyses showed inflection points coinciding with intervention initiation; AUD remained stable pre-intervention but declined consistently afterward, mitigating seasonal influences. The consistent reduction in antimicrobial use across matched quarters enhances confidence that the observed effects may be attributable to the intervention rather than seasonal fluctuations. However, the retrospective quasi-experimental design limits causal inference; we cannot definitively attribute observed effects to specific interventions due to potential confounding factors. No other major initiatives were implemented during the study period, and the microbiology laboratory confirmed unchanged testing methodologies, eliminating detection bias.

The study suggests this AMS strategy feasibility in tertiary hospitals, which typically possess necessary specialists and information technology infrastructure. However, implementation in resource-limited secondary/primary care institutions faces multiple challenges: staffing shortages, infrastructure gaps (incomplete electronic records, inadequate microbiology capacity, unintegrated hospital/lab systems), and limited administrative support. We recommend tiered implementation: full interventions in tertiary hospitals; basic MDT teams in secondary hospitals; and prescription review systems in primary care. For rural areas, we suggest establishing regional AMS networks through medical alliances and incorporating AMS training into rural practitioner continuing education. From a global perspective, this study offers valuable references for resource-limited developing countries.

## Conclusion

This study suggests that implementing this AMS strategy, integrating multidisciplinary administrative, professional, and technical interventions, offers a potentially effective strategy for reducing antimicrobial usage and combating AMR in the tertiary general hospital.

## Limitations

This study has some limitations. First, the single-center design limits generalizability. Although we employed multiple methods to control confounding, residual factors may persist. Future multicenter cluster-randomized trials are needed. Second, as a retrospective quasi-experiment, time-related confounding remains possible despite seasonal adjustment via quarter-matching analyses. The lack of complex causal modeling due to retrospective data could be addressed through prospective multicenter interrupted time series studies, with future analyses incorporating age-stratified, infection type-stratified antimicrobial use assessments and antibiotic category within departments, to establish causal relationships. In future prospective studies, the adjustment of confounding variables (e.g., patient case mix and staffing changes)should also be fully considered. Third, the relatively short study duration precluded long-term feasibility evaluation, and the omission of key outcomes (cost-effectiveness, patient safety metrics) represents important future research directions. Finally, this study was primarily conducted at a tertiary general hospital in China. The feasibility of implementing this approach in other regions and hospitals requires further validation, particularly considering potential limitations in scaling such multidisciplinary interventions across institutions with constrained infrastructure, human resources, or administrative support.

## Data Availability

The raw data supporting the conclusions of this article will be made available by the authors, without undue reservation.
